# Investigating the experiences of older adults with osteoporosis focusing on the diagnostic journey and pathways to specialist care

**DOI:** 10.1186/1472-6955-14-S1-S8

**Published:** 2015-10-08

**Authors:** Margaret Smith

**Affiliations:** 1 Division of Nursing, School of Health Sciences, Queen Margaret University, Edinburgh, EH21 6UU, UK

## Background

Osteoporosis is a worldwide under diagnosed, and under treated disease, with high risk of fracture, and increased morbidity and mortality (IOF 2015) [1]. There have been large advances in diagnosis, management and prevention of secondary fractures (Kanis et al 2013) [2], (McLellan et al 2004) [3], but in many cases access to specialists is delayed due to healthcare staff having limited awareness of osteoporosis.

## Aims

To investigate the experiences of older adults with osteoporosis focusing on the diagnostic journey and pathways to specialist care.

## Methods

A qualitative research design informed by interpretivism was devised. Participation was voluntary. Semi-structured interviews were undertaken with people [age 64+] with a confirmed diagnosis of osteoporosis, mainly in participants' homes. Data analysis: Line by line coding was used, descriptive codes attached, hierarchies developed, relationships viewed and patterns within and across cases examined using computer aided qualitative data analysis. The preliminary coding framework was thus refined. Data collection was progressed towards data saturation.

## Results

A purposive sample of 16 people (age range 64 to 85 years) were interviewed from March to December 2012. Themes identified included the diagnostic journey and living with osteoporosis. Access to specialist osteoporosis services were either patient or healthcare professional initiated, and included cases of multiple fractures, single fracture, silent presentation (osteoporosis confirmed during other treatments or due to referral from primary care as risk factors recognised).

## Conclusions

Problems are evident in the diagnostic journey for older people with osteoporosis. Improved knowledge of osteoporosis, better use of epidemiological data and skilled systematic assessment are needed.
